# Caspase-3 over-expression is associated with poor overall survival and clinicopathological parameters in breast cancer: a meta-analysis of 3091 cases

**DOI:** 10.18632/oncotarget.23667

**Published:** 2017-12-23

**Authors:** Xia Yang, Da-Ni Zhong, Hui Qin, Pei-Rong Wu, Kang-Lai Wei, Gang Chen, Rong-Quan He, Jin-Cai Zhong

**Affiliations:** ^1^ Department of Pathology, First Affiliated Hospital of Guangxi Medical University, Nanning, Guangxi Zhuang Autonomous Region 530021, China; ^2^ Department of Chemotherapy, Tumor Hospital of Guangxi Medical University, Nanning, Guangxi Zhuang Autonomous Region 530021, China; ^3^ Department of Medical Oncology, First Affiliated Hospital of Guangxi Medical University, Nanning, Guangxi Zhuang Autonomous Region 530021, China

**Keywords:** caspase-3, breast cancer, prognosis, clinicopathological features, meta-analysis

## Abstract

Caspase-3 is a vital executioner molecule during the apoptotic process. Numerous studies have revealed the close association of caspase-3 expression and breast cancer. Nevertheless, the prognostic value of caspase-3 expression for patients with breast cancer remains uncertain. To thoroughly analyze the prognostic effect of caspase-3 expression on the clinicopathological features and survival of breast cancer, we conducted this meta-analysis. With various search strategies, electronic databases were comprehensively searched. A total of 3091 patients from 21 studies were ultimately obtained. The analysis results indicated that increased expression of caspase-3 had a negative influence on the overall survival (OS) of breast cancer (HR = 1.73, 95%CI 1.12–2.67, *P* = 0.014). Subgroup analyses based on race revealed that the value of caspase-3 for evaluating patients’ OS was more useful in Asian patients (HR = 3.16, 95%CI 1.20–8.15, *P* = 0.020), and subgroup analyses based on study analytical methods revealed that caspase-3 was a risk factor for breast cancer patients in multivariate overall survival analyses (HR = 1.67, 95%CI 1.02–2.75, *P* = 0.044). As for the relationship between caspase-3 expression and breast cancer subtype as well as progression, caspase-3 might serve as a risk factor for the progestogen receptor (PR) and human epidermal growth factor receptor-2 (HER-2) subtypes (OR = 1.44, 95%CI 1.09–1.89, *P* = 0.010; OR = 1.76, 95%CI 1.18–2.62, *P* = 0.050, respectively) of breast cancer. However, no evidence showed that increased expression of caspase-3 was statistically correlated with tumor differentiation state (low/moderate or high), tumor TNM stage (I-II/III-IV) or lymph node metastasis (–/+). In conclusion, this meta-analysis revealed that increased caspase-3 expression was significantly associated with worse prognosis and two subtypes of breast cancer. More prospective studies are urgently needed to define the prognostic value of caspase-3 expression in patients with breast cancer.

## INTRODUCTION

Breast cancer, which adversely affects women’s physical and psychological health, has the highest incidence of female malignant tumors. It is reported that approximately 1.7 million newly diagnosed cases and 521,900 deaths occurred on a global scale in 2012 [1]. Breast cancer is usually divided into ductal or lobular carcinoma according to location, and invasive ductal carcinoma (IDC) is the most common type. Although the diagnosis and treatment techniques have improved greatly, patient mortality remains high because of chemotherapy resistance and distant metastases. Therefore, it is necessary to identify a more valuable and convenient biomarker that can be tested in early breast cancer, then used to reduce the disease mortality.

Several studies have reported that breast cancers with a high apoptosis index have a better prognosis than those with lower or absent levels of apoptosis [2–5]. Some studies also showed that apoptosis factors were over-expressed in advanced breast cancer [6, 7]. Caspase-3, the central member of the cysteine-aspartic acid protease (caspase) family, was found to play a dominant role in the apoptotic signaling pathway and to regulate cellular apoptosis. Poly (ADP-ribose) polymerase 1 (PARP-1) is responsible for DNA repair and programmed cell death and is the most important substrate of caspase-3. During the early stages of apoptosis, caspase-3 is cleaved into 29- and 85-kDa fragments [8] by PARP-1. Furthermore, cleavage of caspase-3 was shown to mediate tumor repopulation in apoptotic tumor cells [9]. The change of caspase-3 expression is related to the carcinogenesis and progression of many tumors, such as colon cancer [10], cervical adenocarcinoma [11], and glioma [12], indicating that caspase-3 level may be a useful biomarker for these tumors.

Increasing evidence has shown that down-regulation of caspase-3 is correlated with the development of breast cancer [6, 7, 9, 22, 23, 29, 31, 38]. Several studies reported that caspase-3 expression decreased the likelihood of developing breast cancer [23, 28], while other studies reached the opposite conclusion [13, 31]. Likewise, several studies indicated that caspase-3 expression was significantly correlated with prognosis in breast cancer patients [7, 35, 44], while some studies found the opposite [6, 33]. To explore the clinicopathological and prognostic value of caspase-3 expression in patients with breast cancer, a meta-analysis was performed in the current study.

## MATERIALS AND METHODS

### Search strategy and study identification

A comprehensive literature search was conducted using the PubMed, Embase, Wiley, Web of Science, ScienceDirect, Wanfang, Chongqing VIP, CNKI and Chinese Biology Medicine databases. All the databases were last updated on July 31, 2017. The search terms were as follows: “breast OR mammary” AND “caspase-3 OR caspase 3 OR casp 3 OR CC3 OR CPP32” AND “cancer OR carcinoma OR tumor OR neoplasm OR sarcoma OR malignan*”. At the same time, we modified the search strategies appropriately to meet the various demands and rules of the different electronic databases. To identify additional studies, we manually searched review articles and bibliographies.

Studies were considered eligible if they met the following criteria: (i) Studies that demonstrated caspase-3 expression in breast cancer tissues; (ii) Studies that evaluated the relationship between caspase-3 expression and clinicopathological parameters, progress or prognosis of breast cancer; (iii) The language of publications was English or Chinese; (iv) Studies that provided sufficient information to assess the hazard ratios (HR) of overall survival (OS), disease-free survival (DFS), post-relapse survival (PRS) or relapse-free survival (RFS), or to evaluate odds ratios (OR) associated with clinicopathological features and their corresponding confidence intervals (CI). If the data were not provided directly, the value had to be inferable from adequate clinic data and/or the reported tables; (v) If multiple publications were found representing the same data, or were written by the same authors, the most informative and most recent publications were selected.

Certain studies were eliminated from the analysis: (i) studies that only offered abstracts or contained limited information, such as letters, reviews, conference abstracts, case reports, editorials, and expert opinions; (ii) studies that had no full text available; (iii) studies that did not contain groups to compare; (iv) studies that were based on cell lines or animals; and (v) studies that contained no information on prognosis, or from which HR and the corresponding CI of prognosis could not be calculated from the supplied data.

### Data extraction

All the eligible studies were carefully reviewed and then the information was extracted from the studies independently by two investigators (Pei-rong Wu and Hui Qin). Discrepancies were settled by the third investigator (Xia Yang), and agreements were reached through discussion, when necessary. For the included studies, the following parameters were obtained: first author’s name; year of publication; country in which the research was performed; number of patients in the study; the cut-off value for caspase-3 positivity; histological cancer types included in the study; caspase-3 assessment methods used, patient follow-up time, univariate/multivariate analysis methods; DFS, RFS, PRS, and OS levels with HRs and corresponding CIs; and clinicopathological parameters. If not specified in the articles, HRs and 95%CIs were calculated from the available data or assessed from Kaplan-Meier survival curves with the methods reported by Tierney et al and Parmar et al. [14]. Since it is a manual operation, to improve the quality of our data, HRs and 95%CIs were assessed from Kaplan-Meier survival curves independently by three investigators, each investigator extracted at least twice on each Kaplan-Meier survival curves, and then took the average.

### Quality assessment

The Newcastle-Ottawa quality assessment scale (NOS) was used to determine the quality of each study [15]. Eight methodology characteristics, constituting three categories including selection, comparability and outcome, were evaluated, and the quality of each study was presented via scores. In NOS, each item could be awarded up to one point, except comparability, which was applied a maximum of two points. Cumulatively, each study can achieve a maximum of nine points. Generally, articles with six points or more were considered as high quality.

### Statistical analysis

Initially, three separate analyses were performed to investigate the effect of caspase-3 on OS, DFS/RFS and clinicopathological parameters. For OS and DFS/RFS, HRs and their corresponding 95%CIs were used to measure the correlation between caspase-3 expression and breast cancer prognosis. Combinations of ORs and 95%CIs were applied to analyze the relationship between high expression of caspase-3 and clinicopathological features including differentiation grade (low/ moderate or high differentiated), lymph node metastasis (–/+), Tumor TNM stage (I–II/III–IV), ER status (–/+), PR status (–/+), and HER-2 status (–/+). We pooled statistical variables directly if values were obviously available, and we used the methods recommended by Parmar1 if the results could not be determined from the data provided. If authors provided Kaplan-Meier graphs instead of showing HRs directly, Engauge Digitizer version 4.1 (http://digitizer.sourceforge.net/) was used to extract values from the Kaplan-Meier curves. Fixed-effects models or random-effects models were used to combine HRs depending on Cochran’s Q tests (Chi squared test; Chi^2^) and inconsistency statistics (I^2^) [16]. If *P* < 0.05 or I^2^ > 50%, indicating significant study heterogeneity, a random-effects model was selected; if not, a fixed-effects model was preferentially used. The values of *p* were determined by a two-sided test. If HR or OR > 1 and the 95%CI was not over 1 (which would suggest a worse outcome associated with high caspase-3 expression in breast cancer), the data were considered statistically significant. Subgroup analyses, which were conducted to eliminate any effects from study heterogeneity, were carried out based on the original characteristics of the studies. We tested for publication bias by applying Begg’s funnel plot and looking for asymmetry [17]. Moreover, owing to the influence of certain low-quality articles, sensitivity analysis was performed by sequentially removing each single study and re-examining the reliability of the results. Stata version 12.0 (Data Analysis and Statistical Software, version 12) was used to carry out these statistical analyses.

## RESULTS

### Identification of usable studies

**Figure 1:**
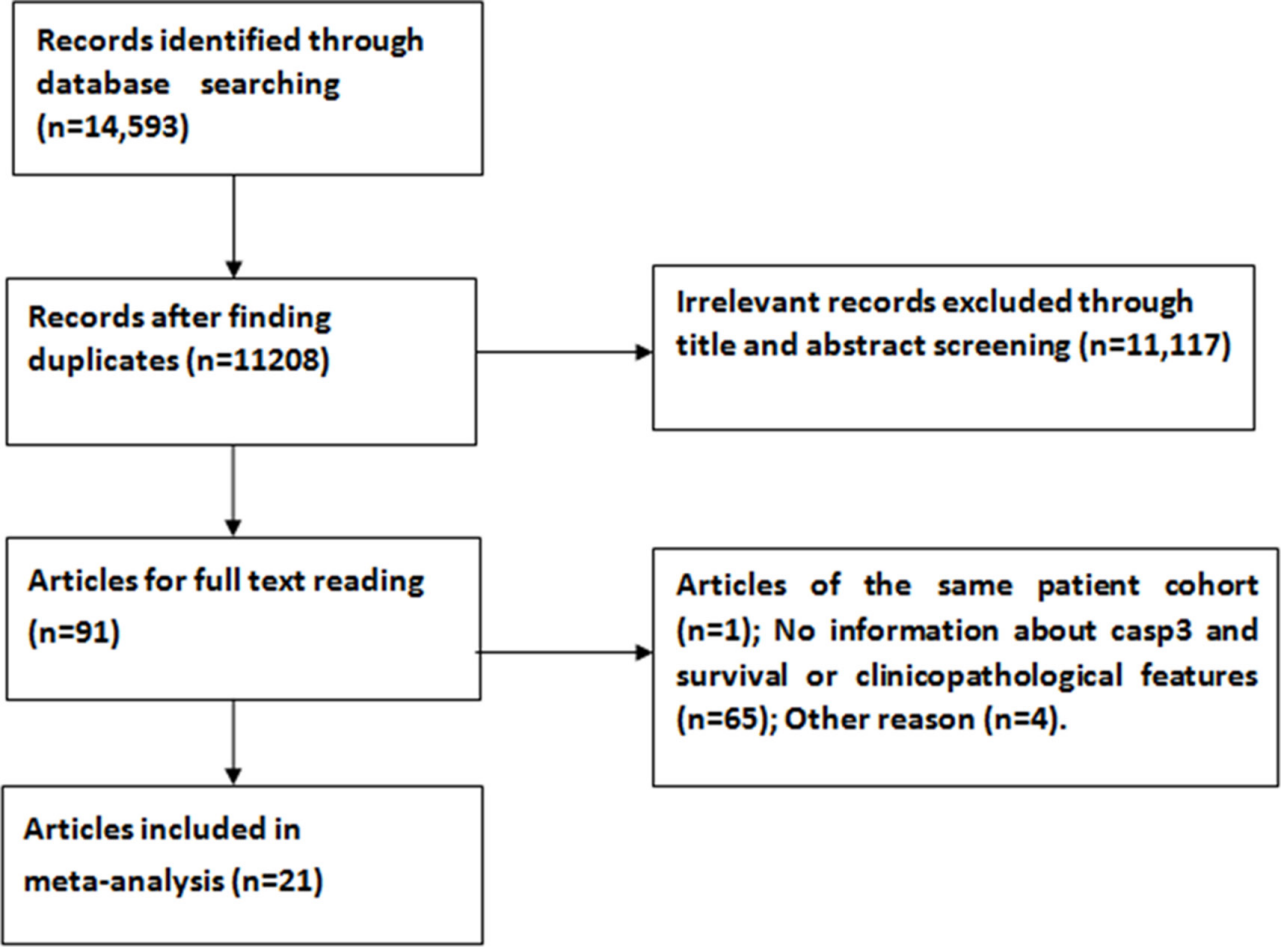
Flow diagram summarizing the process of study selection

With the aforementioned search strategies in electronic databases, 92 eligible studies were identified initially. By scrutinizing titles and abstracts, we excluded 71 articles during the final check because three of them included the same sample population and the remaining were obviously lacking sufficient survival data or clinicopathological features and therefore did not meet our inclusion criteria. After reviewing the full papers, a total of 21 records were ultimately obtained for this meta-analysis [6, 7, 9, 18–37]. One further study [38] was excluded because it contained a duplicate population. The details of the article inclusion and exclusion criteria are shown in Figure 1.

### Study characteristics and study methodological quality

Twenty-one studies met the inclusion criteria, with a range from 31 to 822 patients per study. The published years of all the studies were between 2001 and 2015. Additionally, among the included studies, 12 were conducted in China, two were carried out in the USA, and the rest originated from Spain, Greece, France, Netherlands, Sweden, Korea and the UK. The caspase-3 status in tissue samples was determined by immunohistochemical (IHC) staining in 18 studies, and real-time polymerase chain reaction (RT-PCR) technology was used in two articles. However, one of the included studies did not mention the precise testing method. Of the 21 studies, nine reported prognostic effect values. Eight of them concentrated on the relationship between caspase-3 expression and OS. Two provided the DFS and RFS rates. Only one offered information about PRF. In addition, four prognostic studies provided the HRs and 95%CI from multivariate analyses. Regarding the methodological quality of all studies, only two of them received scores of five or lower, which suggested they were of relatively low quality. The remaining 19 articles all had scores of six or higher, suggesting better methodological quality. The main characteristics of the 21 enrolled studies are summarized in Table 1.

**Table 1:** Characteristics of the included studies

Author	Year	Country	No. of patients	Cut-off value for caspase-3	The level of caspase-3 in breast cancer	Histological Types	Method	Follow-up (Months)	Multia	Outcome	HR(95%CI)	Risk evaluation methods	Quality score
Blazquez S	2006	Spain	206	< 10%	decreased	IDC	IHC	70.86^*^	NO	OS	0.971(0.457–2.041)	DE	8
Nakopoulou L	2001	Greece	137	< 20%	increased	IDC,ILC	IHC	70.34^**^	YES	OS	3.309(1.1276–9.7155)	Reported	7
Zhou L	2013	China	119	< 10%	increased	various	IHC	66^*^	YES	OS	5.307(2.001–14.075)	Reported	7
Vegran F	2008	France	130	NA	increased	IDC	RT-PCR	138^*^	YES	OS,DFS	OS:1.11(0.61–2.04) DFS:1.04(0.61–1.79)	Reported	7
Engels CC	2013	Netherlands	822	< 0.49	increased	Early BC	IHC	120^*^	YES	OS,RFP, CPP	OS:0.984(0.669–1.447) RFP:1.865(0.849–4.099)	Reported	7
Nassar A	2008	USA	91	< 10%	decreased	NA	IHC	120	NO	OS,DFS, CPP	OS:1.75(0.24–13.00) DFS:2.63(0.18–38.25)	Reported	6
Tobin NP	2014	Sweden	111	NA	decreased	various	NA	120	NO	PRS	0.37(0.22–0.59)	Reported	7
Huang Q	2009	China	297	< 10%	increased	NA	IHC	120	NO	OS, RFS	OS: 5.29(1.70–16.46) RFS: 2.33(1.53–3.56)	Reported	7
Li LH	2012	China	180	< 25%	decreased	various	IHC	60	YES	OS	1.519(1.068–2.493)	Reported	7
Wang HM	2012	China	66	< 10%	decreased	Basal-like	IHC	-	-	CPP	-	Reported	8
Hei JY	2010	China	96	< 5%	decreased	NA	IHC	-	-	CPP	-	Reported	7
Yang XF	2008	China	95	< 5%	decreased	various	IHC	72^*^	-	CPP	-	Reported	6
Devarajan E	2002	USA	31	NA	decreased	various	RT-PCR	-	-	CPP	-	Reported	5
Grigoriev MY	2015	UK	60	< 25%	increased	various	IHC	-	-	CPP	-	Reported	6
Ma ZS	2005	China	60	< 10%	decreased	various	IHC	-	-	CPP	-	Reported	5
Sui WY	2010	China	277	< 10%	decreased	NA	IHC	-	-	CPP	-	Reported	7
Hu HH	2007	China	45	< 25%	increased	NA	IHC	-	-	CPP	-	Reported	7
Wu MH	2012	China	67	< 25%	increased	IDC	IHC	-	-	CPP	-	Reported	8
Wang XM	2011	China	71	< 10%	decreased	IDC	IHC	-	-	CPP	-	Reported	8
Zhong GS	2012	China	60	< 10%	decreased	IDC	IHC	-	-	CPP	-	Reported	7
Xue SX	2011	China	70	< 10%	decreased	IDC	IHC	-	-	CPP	-	Reported	7

Abbreviations: ^a^multivariate analysis; ^*^median; ^**^mean; BC: breast cancer; IHC: immunohistochemistry; NA: not available; HR: hazard ratio; OS: overall survival; RFS: relapse-free survival; DFS: disease-free survival; DE: data extrapolated; IDC: invasive ductal breast carcinoma; ILC: invasive lobular breast carcinoma; CPP: clinicopathological parameter; PRS: post-relapse survival.

### Meta-analysis

#### Association between caspase-3 expression and OS

**Figure 2:**
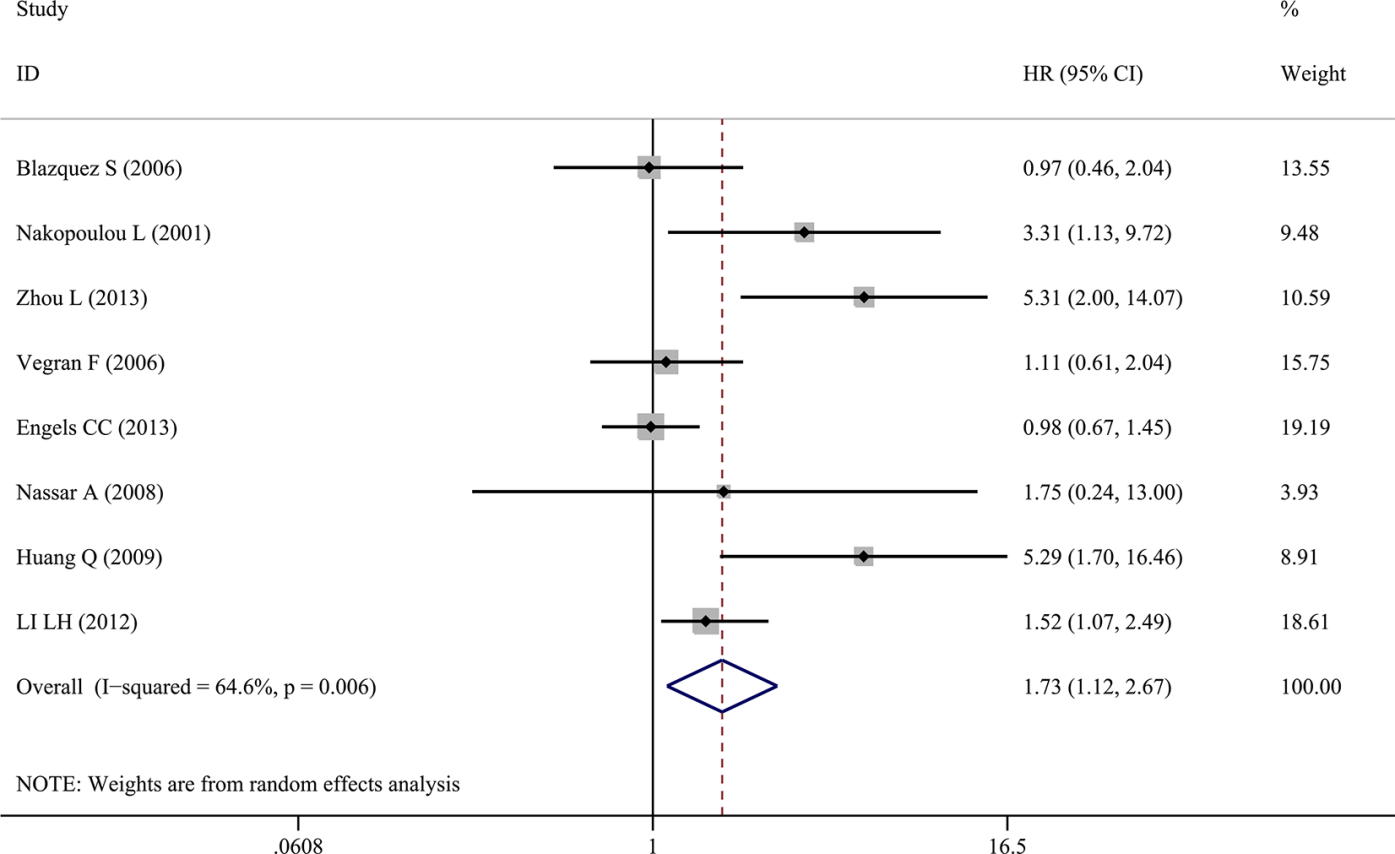
Forest plots of merged analyses of overall survival (OS) and expression of caspase-3 Overall survival (random-effects model): The results indicated increased caspase-3 had a worse influence on OS (HR = 1.73, 95%CI 1.12–2.67, *P =* 0.014).

**Figure 3:**
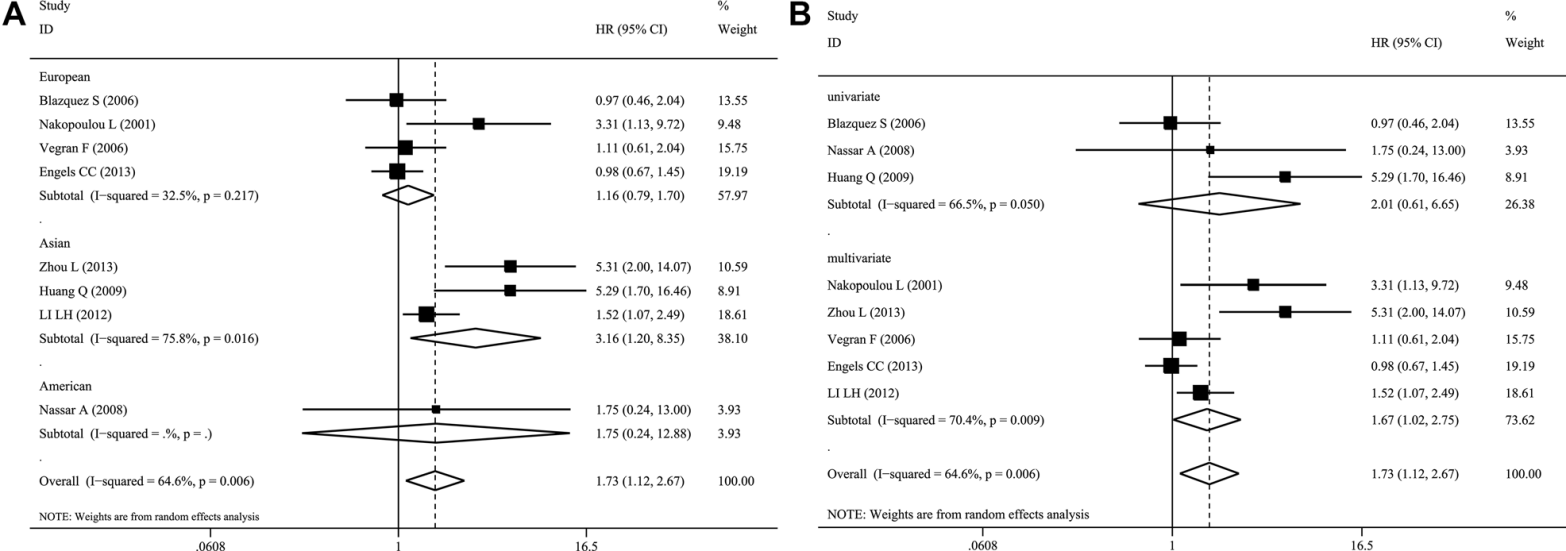
Forest plots of merged analyses of OS and expression of caspase-3 in different subgroups (**A**) Ethnicity subgroup: The results revealed that high caspase-3 expression was significantly associated with poor OS in the Asian subgroup (HR = 3.16, 95%CI 1.20–8.35, *P =* 0.020), (**B**) Analytical methods subgroup: The results revealed that caspase-3 was a risk factor for breast cancer patients using multivariate overall survival analysis (HR = 1.67, 95%CI 1.02–2.75, *P =* 0.044), but no statistical significance was found using univariate subgroups (HR = 2.01, 95%CI 0.61–6.65, *P =* 0.254).

**Figure 4:**
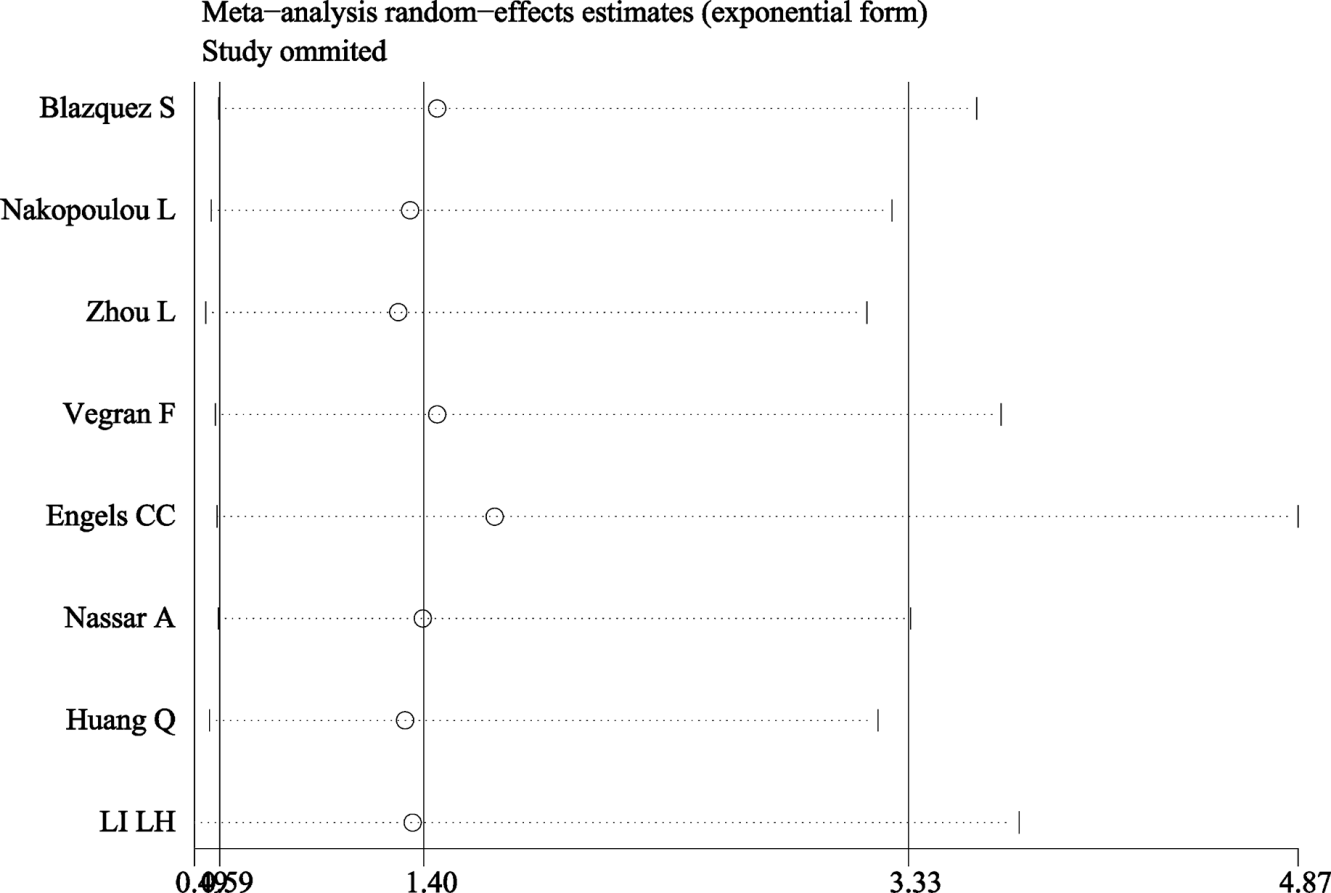
Sensitivity analysis of OS Sensitivity analyses showed that removing the article of Engels CC et al, altered the results.

A total of eight studies were included evaluating the relationship between caspase-3 expression and OS in breast cancer (Figure 2). The study heterogeneity (χ^2^ = 19.78, *P =* 0.006, I^2^ = 64.6%) was taken into consideration. Therefore, we combined the HRs using a random-effects model. The value of the pooled HRs was 1.73 (95%CI 1.12–2.67, *P =* 0.014), indicating that increased caspase-3 levels had a negative influence on the OS of breast cancer. Given the observation of obvious study heterogeneity (*P =* 0.006, I^2^ = 64.6%), stratified analyses were conducted based on similar features. Subgroup analyses were conducted according to ethnic group in fixed-effects models and random-effects models successively because significant study heterogeneity (*P =* 0.016, I^2^ = 75.8%) was observed in Asian patients but absent in European patients (*P =* 0.217, I^2^ = 32.5%). The results showed that up-regulated caspase-3 was mainly correlated with poor OS in the Asian group (pooled HR = 3.16, 95%CI 1.20–8.35, Figure 3A). However, statistical significance was not found in the European group. In addition, subgroup analyses based on study analytical methods revealed that caspase-3 was a risk factor for breast cancer patients using a multivariate overall survival analysis (HR = 1.67, 95%CI 1.02–2.75, *P =* 0.044) (Figure 3B). Since subgroup analysis couldn’t reduce the study heterogeneity from merging the OS values, we inferred that the study heterogeneity may affect multiple parameters, so sensitivity analysis was performed by sequentially removing a single study from the analysis and re-examining the result. This analysis altered in the results somewhat (Figure 4). Sensitivity analysis revealed that when the article of Engels CC et al. was excluded, study heterogeneity was reduced but the I^2^ statistic was still larger than 50% (*P =* 0.021, I^2^ = 59.7%). It may be that this study contained many samples (575 patients) and thus carried a greater influence on the results from the combination of HRs and their corresponding 95%CI. In addition, other factors, such as methods, the cut-off value for caspase-3 positivity and the qualities of each study, may contribute to the overall study heterogeneity.

#### Association between caspase-3 expression and DFS as well as RFS

Only two of the included studies, those reported by Vegran F et al. [6] and Nassar A et al. [30], investigated the relationship between caspase-3 level and breast cancer DFS. Therefore, the pooled HRs of DFS were not calculated. Vegran F and colleges measured caspase-3 expression by reverse transcription-PCR in 130 invasive ductal breast carcinomas and found that caspase-3 levels were higher in carcinoma tissues than in corresponding non-neoplastic tissues, but up-regulation of caspase-3 had no significant association with DFS (HR = 1.04, 95%CI 0.61–1.79). The other group (Nassar A et al.), however, investigated caspase-3 expression in 91 breast cancers by immunohistochemistry and found that caspase-3 levels were reduced in carcinoma tissues and correlated with tumor grade, but this had no significant correlation with OS or DFS in patients.

Likewise, only two articles reported the relationship between caspase-3 levels and RFS rates in breast cancer. Research conducted on 297 Chinese patients by Huang Q. et al. [9] suggested that up-regulation of caspase-3 was related to relapse-free survival in patients with breast carcinoma. Meanwhile, the other group (Engels CC. et al.) investigated caspase-3 expression in 822 Netherlands patients and revealed no significant correlation between caspase-3 expression and patients’ RFS. We concluded that caspase-3 expression may vary by race, but more evidence is needed to confirm this finding.

In particular, one study mentioned post-relapse survival (PRS) and showed that down-regulation of caspase-3 played a significant, negative role in PRS. However, because it was only one study and because of the small sample size (111 patients), this conclusion needs further investigation to be validated.

#### Association between caspase-3 expression and clinicopathological parameters

**Figure 5:**
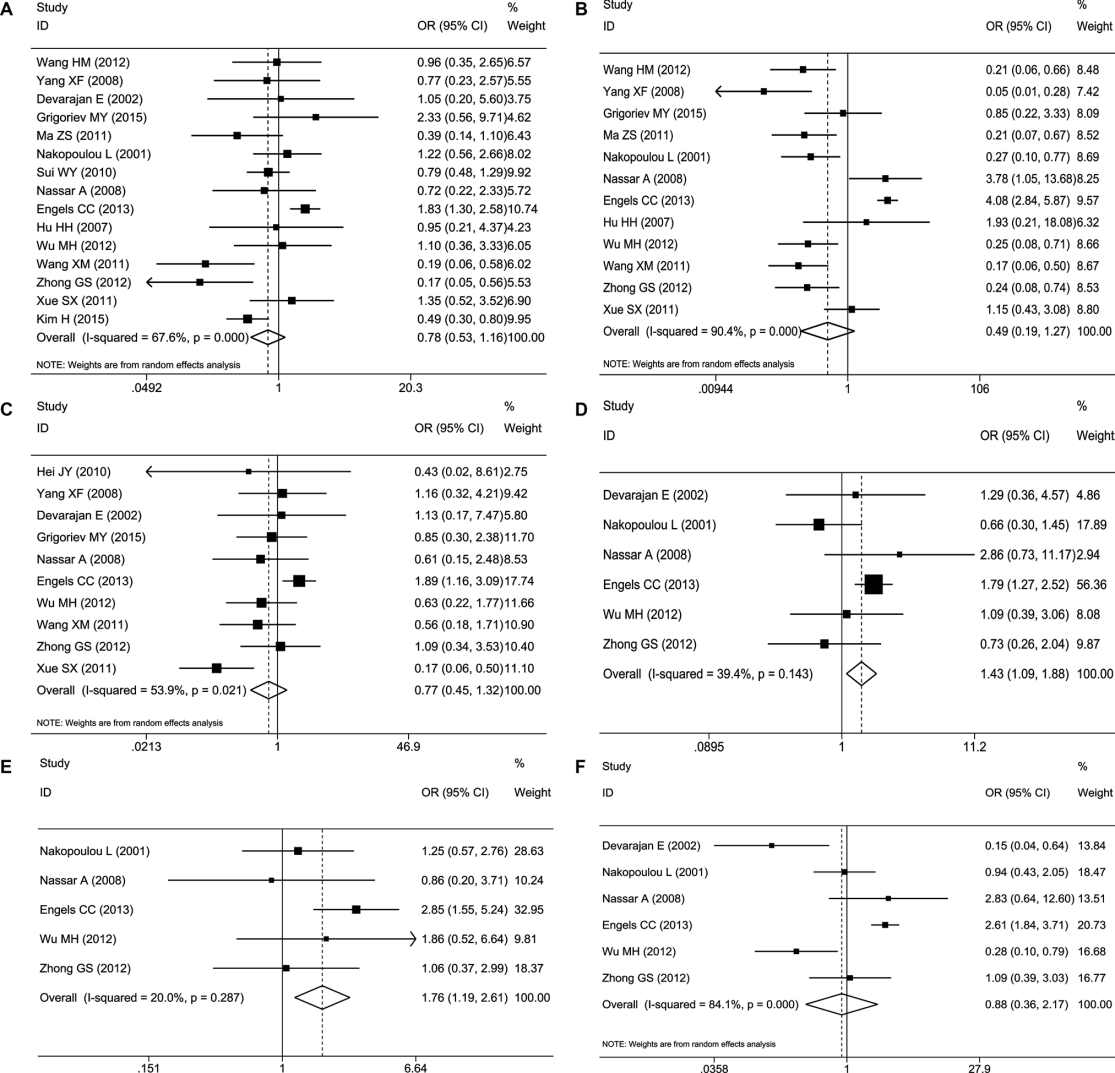
Forest plots of merged analyses of different clinicopathological features and expression of caspase-3 (**A**) Lymph node metastases (–/+) (random-effects model): No evidence showed that high caspase-3 expression was significantly correlated with lymph node metastases (–/+) (OR = 0.78, 95%CI 0.53–1.16), (**B**) Tumor differentiation grade (low, moderate or high) (random-effects model): No evidence showed that high caspase-3 expression was significantly correlated with tumor differentiation grade (low, moderate, or high) (OR=0.49, 95%CI 0.19–1.27), (**C**) Tumor TNM stage (I–II/III–IV) (random-effects model): No evidence showed that high caspase-3 expression was significantly correlated with tumor TNM stage (OR = 0.77, 95%CI 0.45–1.32), (**D**) PR status (–/+) (fixed-effects model): Evidence revealed a significant correlation between increased caspase-3 levels and PR status (–/+) (OR = 1.44, 95%CI 1.09–1.89, *P =* 0.010), (**E**) HER-2 status (–/+) (fixed-effects model): The results suggested that increased expression of caspase-3 was significantly associated with HER-2 positive status (OR = 1.76, 95%CI 1.18–2.62, *P =* 0.050), (**F**) ER status (–/+) (random-effects model): No evidence showed that high caspase-3 expression was significantly correlated with ER status (–/+) (OR = 0.88, 95%CI 0.36–2.16).

Sixteen studies were included in this meta-analysis because they included clinicopathological parameters. Fifteen studies comprising 1042 patients studied the association between caspase-3 expression and lymph nodes metastases. Twelve studies comprising 866 patients measured the correlation of caspase-3 levels with tumor differentiation grade. Ten studies comprising 672 patients reported the relationship between caspase-3 expression and Tumor TNM stage. However, no evidence showed that up-regulation of caspase-3 expression was correlated with lymph node metastasis (–/+) (OR = 0.78, 95%CI 0.53–1.16, Figure 5A), tumor differentiation grade (low, moderate or high) (OR = 0.49, 95%CI 0.19–1.27, Figure 5B) or tumor TNM stage (OR = 0.77, 95%CI 0.45–1.32, Figure 5C). Random-effects models were adopted for the risk assessment of all three clinicopathological parameters (tumor differentiation grade, lymph nodes metastases and Tumor TNM stage) with caspase-3 because of the high study heterogeneity (*P =* 0.00, I^2^ = 90.4%; *P* < 0.001, I^2^ = 67.6%; *P =* 0.021, I^2^ = 53.9%, respectively). Additionally, there were also several studies that calculated the relation between caspase-3 expression and ER status (–/+) (six studies with 576 patients), PR status (–/+) (six studies with 584 patients) and HER-2 status (–/+) (six studies with 403 patients). Intriguingly, the pooled OR of caspase-3 expression with PR status (–/+) was 1.44 (95%CI 1.09–1.89, *P =* 0.01, Figure 5D), and with HER-2 status (–/+) was 1.76 (95%CI 1.18–2.62, *P =* 0.05, Figure 5E), which suggested that increased expression of caspase-3 was significantly associated with PR- and HER-2-positive subtypes. However, the pooled OR of ER status with caspase-3 expression revealed that no significant differences in caspase-3 expression were observed between carcinoma tissues and normal controls (OR = 0.88, 95%CI 0.36–2.16, Figure 5F). The main clinicopathological parameters taken from the enrolled studies are summarized in Table 2.

**Table 2:** Pooled OR and 95%CI from meta-analysis of clinicopathological parameters

	Number of results	Number of patients	OR(95%CI)	*P* value	Heterogeneity			Model used
					*I* ^2^	*P*	*I* ^2^ (%)	
Differentiation (low, moderate or high)	12	866	0.492 (0.190–1.275)	0.144	114.77	0.000	90.4	REM
lymph nodes metastases (–/+)	15	1,042	0.782 (0.528–1.160	0.222	43.24	0.000	67.6	REM
TNM stage (I–II/ III–IV)	10	672	0.773 (0.453–1.320)	0.346	19.54	0.021	53.9	REM
ER status (–/+)	6	576	0.878 (0.356–2.167)	0.778	31.52	0.000	84.1	REM
PR status (–/+)	6	584	1.431 (1.090–1.880)	0.010	8.25	0.143	39.4	FEM
HER-2 status (–/+)	5	403	1.762 (1.191–2.607)	0.05	5	0.287	20	FEM

Abbreviations: OR: odds ratio; REM: random-effects model; FEM: fixed- effects model.

#### Publication bias

**Figure 6:**
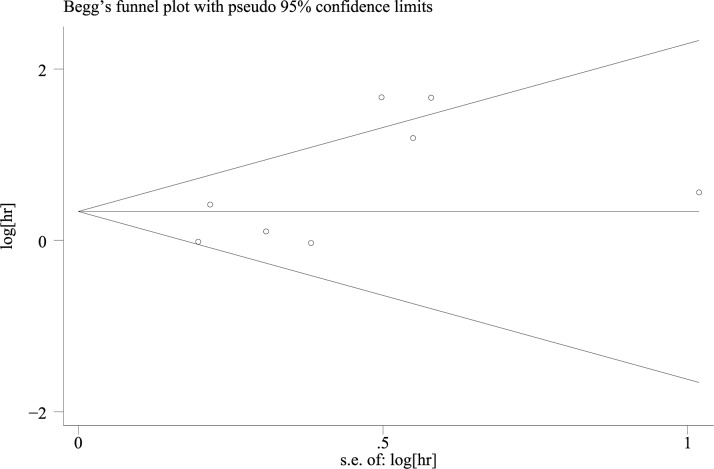
Begg’s funnel plot of publication bias for OS: There was no evidence of asymmetry in the funnel plots for OS

**Figure 7:**
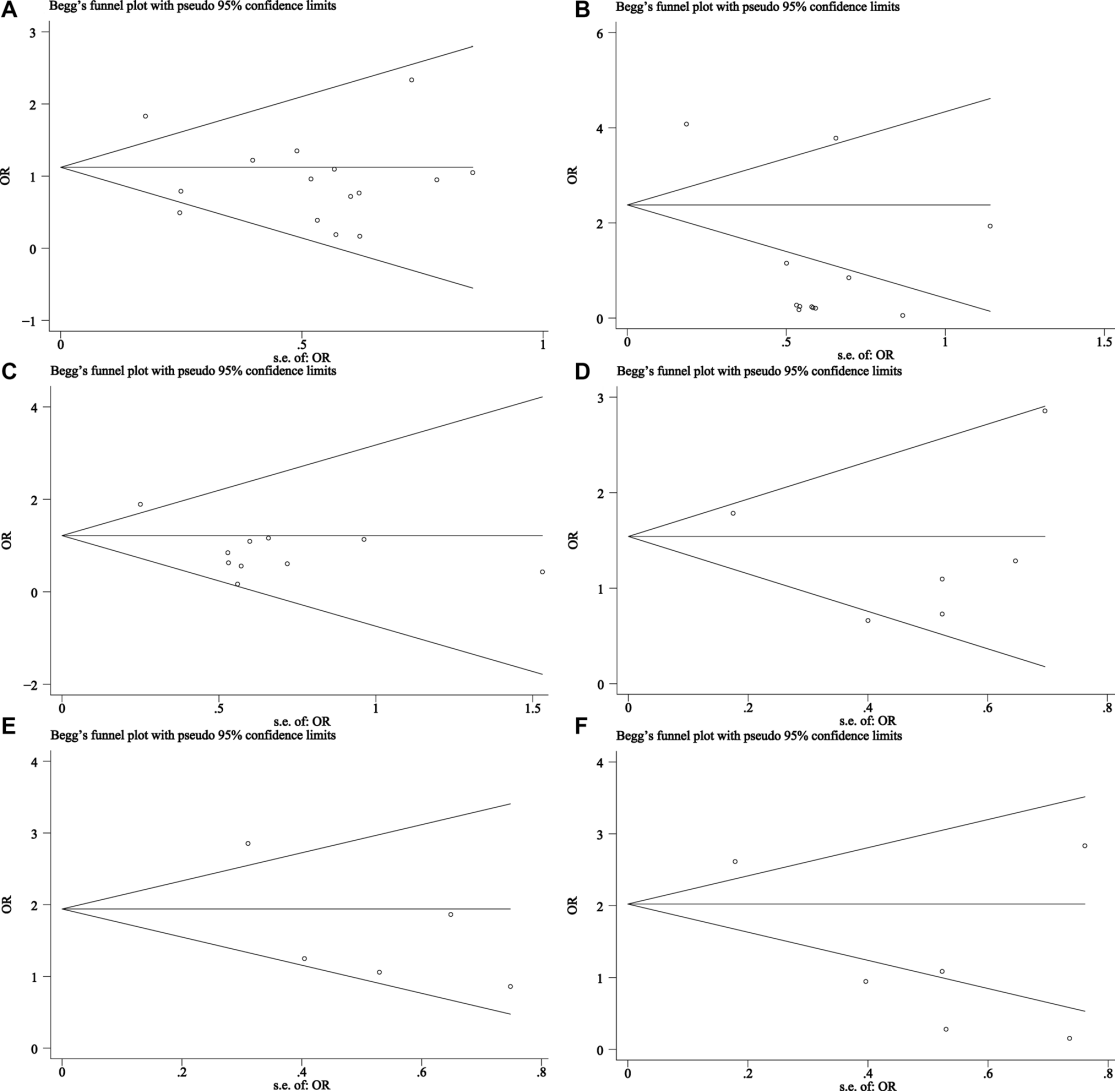
Begg’s funnel plot of publication bias for clinicopathological features (**A**) Lymph nodes metastases (–/+) (random-effects model), (**B**) Tumor differentiation grade (low, moderate or high differentiated) (random-effects model), (**C**) Tumor TNM stage (I–II/III-IV) (random-effects model), (**D**) PR status (–/+) (fixed-effects model), (**E**) HER-2 status (–/+) (fixed-effects model), (**F**) ER status (–/+) (random-effects model): The results indicated a slight bias in tumor differentiation grade and ER status with their *P* values being 0.005, 0.042, respectively.

Begg’s funnel plot was used to estimate the publication bias of these studies. There was no remarkable evidence of asymmetry in the funnel plots for OS (As shown on Figure 6), whereas analysis of the publication bias in clinicopathological parameters indicated that bias could be found in level of tumor differentiation and ER status, with *P v*alues of 0.005 and 0.042, respectively (Figure 7).

## DISCUSSION

Apoptosis, or normal programmed cell death, is a biological process for clearing out senescent and abnormal cells. Therefore, it is generally believed that apoptosis dysregulation may promote the pathogenesis and progression of tumors [39, 40] Caspase-3, activated directly by caspase-8, -9 and the apoptosome, is one of the executor caspase that take part in both extrinsic and intrinsic apoptotic pathways. It is normally primarily found in the cytoplasm, and during the apoptotic process, it is transported into the nucleus to interact with its nuclear substrates [41]. Because of the role of caspase-3 in apoptosis, some researchers suggest that its reduced expression might result in tumor cells escaping from apoptosis and ultimately lead to tumorigenesis and deterioration [36, 42]. However, additional studies [7, 9, 35, 43] reported that caspase-3 was found to be a carcinogenesis promoter in both *in vivo* and *in vitro* studies. For example, a higher level of caspase-3 expression was found in invasive breast cancer versus corresponding normal breast tissue [43, 44], and indicated poor prognosis for breast carcinoma patients. Huang Q et al. [9] reported the surprising discovery that activated caspase-3 played a vital role in tumor cell repopulation and the increased rate of tumor recurrence. Moreover, according to both *in vivo* and *in vitro* experiments from Feng X et al. [45], caspase-3 was also involved in angiogenesis promotion in dying tumor cells after irradiation (HT-29 and HT-29 CASP3DN cells). The research of Liu X et al. indicated that caspase-3 may facilitate genome instability and carcinogenesis in breast cancer cells (MCF10A cells) exposed to radiation [46].

According to the previous studies, whether caspase-3 over-expression or caspase-3 down-regulation was indicative of poor survival in patients with breast cancer remains controversial. Although many published studies have investigated this problem, the lack of large sample sizes made further inquiry difficult. Meta-analysis, a quantitative method integrating multiple individual studies to reach a more reliable conclusion, was used in this study. To explore the correlation between caspase-3 and prognosis as well as between caspase-3 and clinicopathological characteristics in breast cancer patients, we searched the published literature as comprehensively as possible, trying to create a larger sample size to analyze caspase-3 function in breast cancer.

In our meta-analysis, twenty-one studies were included, with nine of them referring to the prognosis (OS, DFS, RFS or RPS) of breast cancer. More than half of the studies suggested the same trend: high caspase-3 expression might be a prospective risk factor for the survival of breast cancer patients. The combined HR gathering from 1982 patients also revealed that up-regulated caspase-3 was remarkably correlated with poor OS of breast cancer patients (HR = 1.73, 95%CI 1.12–2.67), especially in Asian patients. Among all of the collecting patients, 30.1% (596) are Asian patients, 65.3% (1295) are European patients and 4.6% (91) are American. It is easy to find that there are three different race patients are included for the present study, however, the pooled HR of Asian patient (HR = 3.16, 95%CI 1.20–8.35) showed caspase-3 to be a risk factor, the combined HR of European and American revealed the expression of caspase-3 had no significant difference between breast cancer and normal sample (HR = 1.16, 95%CI 0.79–1.70 and HR = 1.75, 95%CI 0.24–12.88,separately). Which indicated that the expression of caspase-3 in different race are probably discrepant and caspase-3 is a prospective risk factor for breast cancer mainly in Asian populations. Subgroup analyses based on study analytical methods revealed that caspase-3 was a risk factor for breast cancer patients with multivariate overall survival analysis (HR = 1.67, 95%CI 1.02–2.75, *P =* 0.044). In addition, 16 studies referring to the relationship between caspase-3 expression and clinicopathological features were included to investigate caspase-3 function, and our pooled results found no evidence that increased caspase-3 was correlated with the lymph nodes metastases (–/+), tumor differentiation grade (low/moderate or high) or tumor TNM stage. However, increased levels of caspase-3 were significantly associated with PR status (–/+) (OR = 1.44, 95%CI 1.09–1.89) and HER-2 status (–/+) (OR = 1.76, 95%CI 1.18–2.62) in breast cancer [46, 47]. Since most of the included studies didn’t include which type of breast cancer they were studying, we could not perform a subgroup analysis based on this factor. However, doing so may reveal more helpful information for clinical diagnosis and treatment.

Recently, a meta-analysis of 12 studies investigated the correlation between caspase-3 expression and the prognosis of patients with digestive tract cancer and failed to show a positive result [39]. In this meta-analysis, we concentrated not only on the prognostic value of caspase-3 in breast cancer but also the clinicopathological significance of this caspase. Overall, we identified a positive result.

Although this meta-analysis aimed to provide the best possible estimate of the correlation between the over-expression and clinical significance of caspase-3 in breast cancer, however, there are still some limitations to our research. First, only eight studies with 1982 patients were included in the meta-analysis of overall survival, the number is still inadequate, and more studies are needed to be included to make the results more credible. Secondly, some survival data are extracted from survival curves, which may less reliable than those directly obtained from the primary studies and introduce subjective bias. Thirdly, we noticed that the study heterogeneity of the combined HRs or ORs of several subgroups were relatively large. The study heterogeneity assessed by using Q statistics in our meta-analysis was significant at *P* < 0.05 and potentially affected our results. Many factors, such as the characteristics of patients (age, ethnic race, subtypes of cancer, clinical stage of cancer, etc.), the cut-off value for caspase-3 positivity, the length of patient follow-up, the adjuvant treatment received by patients, the method of caspase-3 quantitation, etc. all played a part in the study heterogeneity. To explore the sources of study heterogeneity, we conducted subgroup analyses of pooled HRs based on different available factors, including race, the method of caspase-3 quantitation, statistical analyses used, and the cut-off value for caspase-3 positivity. Further analyzed, more than one methods were used to calculate the expression of caspase-3 in the twenty-one included studies, though we have conducted sensitivity analysis and subgroup analysis based on varied parameters, the inconformity of this item may still result in the heterogeneity of overall results. On the other hand, cut-off value of positive caspase-3 expression were varied, this factor may also cause the heterogeneity of pooled results in another way and may be restricted to expand the clinical applicability. Unified detection methods and division criteria of high expression caspase-3 should be established to make it suitable for clinical applications. Meanwhile, the combined HR that described the correlation between caspase-3 and overall survival rate in breast cancer was relatively weak (HR = 1.73). As a matter of experience, an RR less than 2 is considered to lack practical use [13], and this rule was applied to evaluate the HRs in the present study. Overall, we come to the conclusion that race, cancer subtype and the cut-off value for caspase-3 positivity were the main factors involved, given their corresponding heterogeneities. Last, the majority of samples in this study were collected from Asian patients especially patients in China, in our future research, to expand the clinical applicability, a wider sample collection is necessary to avoid the selection bias.

In addition, publication bias, which could create a barrier to identifying all eligible studies, was analyzed. According to Begg’s test for OS, no publication bias was observed (*P* > 0.05). However, publication bias could still not be completely excluded. Although we tried to search the databases as much as possible, qualified studies were missed for various reasons. We could not include unpublished papers, studies that were only provided in abstract form, or those written in languages other than English. In addition, the studies with positive results were more likely to be reported in English, whereas those with non-significant results were prone to be published in their native languages, and thus selection bias was inevitable. Another potential bias was derived from the methods of HR extrapolation. Most HRs and their 95%CIs were directly obtained from the studies. If the authors did not present these data directly, we extrapolated them by calculating a survival comparison statistic or by having two authors independently extract data from the survival curves. Obviously, the latter method might have been less reliable than the two former methods.

In conclusion, our meta-analysis showed that high caspase-3 expression was significantly associated with a worse prognosis for patients with breast cancer, especially for Asian patients. Moreover, caspase-3 expression was significantly related to the PR and HER-2 status of patients with breast cancer. Therefore, caspase-3 might be a potential biomarker for predicting advanced stage and poor overall survival in breast cancer patients. More clinical studies with larger sample sizes should be performed to reduce the study heterogeneity and further verify this conclusion.
